# A critical analysis of sarcoidosis incidence assessment

**DOI:** 10.1186/2049-6958-8-57

**Published:** 2013-09-03

**Authors:** Jerome M Reich

**Affiliations:** 1 Thoracic Oncology Program, Earle A Chiles Research Institute, 5251 NE Glisan, Bldg A, Portland, OR97213–2967, USA

**Keywords:** Diagnosis, Epidemiology, Etiology, Radiography, Sarcoidosis, Statistics and Numerical data

## Abstract

Valid sarcoidosis incidence assessment is contingent on access to medical care, thoroughness of reportage, assiduity of radiographic interpretation, employment and health care screening policies, misclassification, and population ethnicity. To diminish ambiguity and foster inter-population comparison, the term “sarcoidosis incidence” must be modified to convey the methodology employed in compiling the numerator. In age-delimited cohorts, valid comparison to population incidence requires age adjustment due to the age-dependency of incidence. The “true incidence” of sarcoidosis is a notional concept: more than 90% of cases are subclinical and radiographically inevident. Occupational causal inference based on incidence differential vs. populations has been undermined by methodological differences in ascertainment and computation.

## Background

Assessment of sarcoidosis incidence requires dividing the annual number of newly identified cases (numerator) by the number in the source population (denominator). This apparent simplicity is illusory, for the numerator is a function of ascertainment methodology and ethnic composition.

## Main text

### Ascertainment methodology

Ascertainment depends on access to medical care and thoroughness of reportage. Additionally, it reflects acuteness of recognition, which is influenced by the experience and assiduity of the interpreting radiologist. Sensitivity to subtle hilar adenopathy and pulmonary shadowing, easily overlooked in mass population screening, is enhanced (“ascertainment bias”) by awareness of a putative germane exposure e.g., among World Trade Center disaster responders. Increasingly frequent performance of chest CT and transbronchial mediastinal biopsy may increase ascertainment [1].

Methodological differences are a major impediment to obtaining comparable incidence data. This is illustrated by differential incidence ascertainment in observable (see below) cases. To formalize its meaning, the term “incidence” must be preceded by an adjective defining the operation by which the numerator is acquired, thereby facilitating valid inter-population comparison. Observable cases are divisible into three operationally defined classes, each subsuming the prior class: Cases identified because of symptoms or an incidental chest radiograph are designated “clinically identified.” Addition of cases identified by health or employment screening chest radiographs (which will vary systematically between populations) defines “clinically recognized.” Addition of cases identified by population screening defines “clinically ascertainable.” This classification scheme can be visualized as concentric circles in a Venn diagram.

### Ethnic composition

#### White incidence in representative western populations

The incidence of clinically identified cases peaks at ages 20–49. It varies widely between ethnic and geographic populations; it is far higher in Blacks and Northern Europeans. The annual incidence of clinically identified sarcoidosis in Whites in the U.K is 3.4 [2], and in the U.S., 2.8 [3]. (Cited annual incidence and prevalence figures are given as cases/100,000). In a more recent UK study, Gribbin et al. reported an ethnically unsorted incidence of 5.1 [4]. In a Swedish study, clinically identified cases were more than twice as high —8— and constituted a minority (42%) of clinically ascertainable cases —19— identified by triennial population screening [5]. The latter figure underestimates the clinically ascertainable incidence, for more frequent screening, (by identifying cases with briefer duration), and screening the entire population rather than 64% would have materially increased the numerator. The clinically recognized incidence of 5 in a U.S. population of mixed Northern European ancestry (in which patients frequently received a chest radiograph as a routine component of their periodic physical examination) [6] is somewhat higher than the UK clinically identified figure [2]. A large Danish study reported a clinically identified incidence of 5; population screening tripled that figure to 15 [7]. Sartwell et al. corroborated the tripling effect on incidence of periodic chest radiographic screening by pointing out that only 28% of cases in a US Naval population were identified because of symptoms [8].

In summary, among US Whites, 3 is a plausible provisional estimate of clinically identified sarcoidosis incidence; 4 may be considered an upper bound. The figure is slightly higher in the UK. The clinically identified incidence in Scandinavian populations is 5. The clinically ascertainable incidence is three-fold the clinically identified incidence.

#### Black: white incidence ratio

The clinically identified sarcoidosis incidence in Blacks in the two reporting, ethnically mixed, US health maintenance organizations were 36.4 and 35.5 [3,9]. Because health maintenance organizations provide equal access to health care for all members and employ a centrally maintained record they circumvent the requirement for reportage and the potential access bias described above. The Black/White incidence ratio of 13 is in the range of previous reports [3].

### Diagnostic decision

#### Clinical diagnosis

A requirement for histological verification is not typically stated in sarcoidosis incidence compendia. Clinical experience has verified the accuracy of clinical diagnosis in stage I; Hillerdal et al. accepted a clinical diagnosis of stage II [5].

#### Misclassification

Individuals exhibiting a local or regional granulomatous response to a neoplasm are not infrequently classified erroneously as having sarcoidosis. The proportion of unascertained chronic berylliosis in sarcoidosis registries is unknown. A phenocopy of sarcoidosis, it can escape detection unless careful inquiry into industrial exposure, supported by beryllium-lymphocyte proliferation testing, has been undertaken [10].

### Lifetime prevalence

Lacking a diagnostic gold standard, autopsy prevalence is imprecise. Enumeration is contingent, in part, on the pathologist’s decision whether pulmonary involvement suffices, whether one or more than one organ demonstrating epithelioid granulomatous changes are required for confirmation, and whether historical or pathologic evidence of potentially confounding, co-existent diseases or exposures are present. Hägerstrand and Linell in a Swedish autopsy series of 6,706 cases reported a prevalence of “certain” (pulmonary or multi-organ epithelioid granulomas) of 640, while the prevalence of uncertain cases was 210. The 640 figure was 10-fold the point prevalence (of clinically ascertained) sarcoidosis estimated by mass radiographic screening in a Stockholm population [11]. Reid reviewed 9,324 forensic autopsies performed in Ohio, in which Blacks constituted one-third, identifying 31 “accepted” cases of sarcoidosis, a prevalence of 333, 10-fold that reported on Cuyahoga death certificates [12]. An additional 11 cases (prevalence 110) were rejected due to possible confounding, principally by illicit drug exposures. Reid noted a ratio of Blacks: Whites of 4.7: 1. Ethnic comparison is limited by small numbers, but this relatively low ratio raises the question whether the reported far higher clinically identified Black incidence might be influenced by a more exuberant response leading to a greater likelihood of clinical recognition. It is not known whether pathological residuals are present in all resolved cases. If not, then post-mortem figures underestimate the lifetime prevalence of sarcoidosis, for the majority of cases resolve. Had we a sarcoidosis marker similar in its simplicity, availability, persistence, sensitivity and specificity to the tuberculin test, the true incidence and lifetime prevalence of sarcoidosis could be ascertained. These figures are incomputable; available figures constitute lower bound estimates.

### Computation

Employing as the denominator the number of persons at risk (rather than the population from which they are derived) in computing the incidence results in a spuriously elevated figure if the peak incidence is concentrated in the age range of the at risk cohort. This consideration is directly applicable to the occupational incidence of sarcoidosis because ~ 85% of cases are identified in the occupational age group, 20–49.

### Causal inference

Several investigators have inferred a causal connection between sarcoidosis and environmental or occupational exposures based on a marked incidence differential vs. population based, clinically identified figures. This inference is weakened by ascertainment bias, lack of an identified putative antigen, the incidence-trebling effect of cohort radiographic surveillance, and the employment of the surveyed, working age cohort as the denominator in the computation [13].

## Conclusions

1. Methodological identity is required to ensure valid inter-population incidence comparison.

2. The clinically identified incidence of sarcoidosis in US Whites is 3; in UK Whites, 3.4; in persons of Scandinavian descent, 5. Population screening of Whites triples that figure. The clinically identified incidence in US Blacks is 36.

3. “The” incidence of sarcoidosis is indeterminable. It is a notional concept due to a combination of ascertainment and methodological contingencies and the preponderance of unobservable cases.

4. Judging from autopsy prevalence, sarcoidosis is common; clinically identified cases constitute a small minority of those that are histologically verifiable.

5. Causal inference based on incidence differential is confounded by differences in case acquisition and incidence computation.

## Competing interests

The author declares that he has no competing interests.

## Authors’ information

The Author has had an active interest in this subject for a number of years

1. Reich JM, Johnson RE: Course and prognosis of sarcoidosis in a nonreferral setting: analysis of 86 patients observed for ten years. *Am J Med* 1985, 78:61–67.

2. Reich JM: Acute myeloblastic leukemia and sarcoidosis: implications for pathogenesis [case report]. *Cancer* 1985, 55:366–369.

3. Reich JM: Sarcoidosis and acute leukemia [letter]. *J Roy Soc Med* 1992, 85:306.

4. Reich JM: Acute myeloblastic leukemia and sarcoidosis [editorial]. *Sarcoidosis* 1993, 10:4–8.

5. Reich JM, Mullooly JP, Johnson RE: Linkage analysis of malignancy-associated sarcoidosis. *Chest* 1995, 107:605–613.

6. Reich JM: Sarcoidosis and agnogenic myeloid metaplasia [case report]. *J Intern Med* 1994, 235:179–182.

7. Reich JM, Johnson RE: Sarcoidosis and malignancy [letter]. *Chest* 1995, 105:1474–1475.

8. Reich JM, Johnson RE: Estimated incidence of clinically identified sarcoidosis in a Northwest United States population. *Sarcoidosis Vasc Diffuse Lung Dis* 1996, 13:173–177.

9. Reich JM: Sarcoidosis mortality in the United States [letter]. *Am J Med* 1997, 102:131.

10. Reich JM: BTS sarcoidosis study [letter]. *Thorax* 1997, 52:102.

11. Reich JM, Brouns MC, O’Connor EA, Edwards MJ: Mediastinoscopy in patients with presumptive stage I sarcoidosis: a risk/benefit, cost/benefit analysis. *Chest* 1998, 113(1):147–153.

12. Reich JM: Racial differences in sarcoidosis incidence: a 5-year study in a health maintenance organization [letter]. *Am J Epidemiol* 1998, 148(1):100–101.

13. Reich JM: Corticosteroid therapy and relapse in sarcoidosis [letter]. *Chest* 1998, 113:559–160.

14. Reich JM: Deciphering histoplasmosis, systemic noncaseating granuloma, and sarcoidosis in the literature [letter]. *Chest* 1998, 113:1143.

15. Reich JM: Malignant neoplasms in pulmonary sarcoidosis [letter]. *Thorax* 1998, 53(7):625–626.

16. Reich JM: How common is sarcoidosis? [letter]. *Sarcoidosis Vasc Diffuse Lung Dis* 1999, 16:108.

17. Reich JM: Sarcoidosis and cancer revisited [letter]. *Eur Respir J* 1999, 14(2) 482–483.

18. Reich JM: Clinical diagnosis of stage I sarcoidosis [letter]. *Chest* 2000, 118(6):1838.

19. Reich JM: Course and prognosis of sarcoidosis in African-Americans vs. Caucasians [letter]. *Eur Respir J* 2001, 17:833.

20. Reich JM: Mortality of intrathoracicsarcoidosis in referral vs. population-based settings: influence of stage, ethnicity, and corticosteroid therapy. *Chest* 2002, 121:32–39.

21. Reich JM: What is sarcoidosis? *Chest* 2003, 124:367–371.

22. Reich JM: Adverse long-term effect of corticosteroid therapy in recent-onset sarcoidosis. *Sarcoidosis Vasc Diffuse Lung Dis* 2003, 20(3):227–234.

23. Reich JM: Sarcoidosis cycle [letter]. *Chest* 2004, 125(3):1172–1173.

24. Reich JM: Eight fundamental unsolved problems in sarcoidosis. *Eur J Intern Med* 2004, 15(5):269–273.

25. Reich JM: Treatment outcome in M.avium pulmonary disease: a correction and comment [letter re. M. avium and sarcoidosis]. *Chest* 2005, 27:1864–1866.

26. Reich JM: Neoplasia in the etiology of sarcoidosis. *Eur J Intern Med* 2006, 17(2):81–87.

27. Reich JM: Investigation of Löfgren’s syndrome [E-letter]. *Chest* 2006 April 12.

28. Reich JM: A requirement for histologic confirmation of stage I sarcoidosis is far more likely to prove harmful than helpful [letter]. *Sarcoidosis Vasc Diffuse Lung Dis* 2007, 24:77.

29. Reich JM: Tissue verification of stage I sarcoidosis [letter]. *Cytopathology* 2007, 18:392–397.

30. Reich JM, Patterson J, Asaph J, Brouns M: Tissue verification of stage I sarcoidosis: the question is if, not how [letter]. *Chest* 2008, 133(6);1529–1530.

31. Reich JM: Concurrent sarcoidosis and lung cancer [letter]. *Chest* 2009; 136:943.

32. Reich JM: Comments on Sarcoidosis (review) [Owen JD et al.] Rapid response *BMJ* Nov 10, 2009.

33. Reich JM: Endobronchial Ultrasonography-Guided Transbronchial Needle Aspiration in Patients With Suspected Sarcoidosis. *Chest* 2010, 137:235–236.

34. Reich JM: Con: The treatment of the granulomatous response is beneficial in acute sarcoidosis [Debate]. *Respir Med* 2010, 104:1778–1781 and 1782–1783. [Pro: Culver DA. Complete debate: 1775–1783.]

35. Reich JM: “Sarcoid like" granulomatous pulmonary disease in World Trade Center disaster responders: Influence of incidence computation methodology in inferring airborne dust causation [letter]. *Am J Ind Med* 2011, 54(9):696.

36. Reich JM: On the nature of sarcoidosis. *Eur J Intern Med* 2012, 23:105–109. doi:10.1016/j.ejim.2011.09.011

37. Reich JM: Sarcoidosis and World Trade Center Disaster [letter]. *J Occup Environ Med* 2012, 54(1):2.

38. Reich JM: Sarcoidosis mortality [letter—in response to Swigris and Baughman]. *Am J Respir Crit Care Med* 2012, 185(4):461–462.

39. Reich JM: Shortfalls in imputing sarcoidosis to occupational exposures [editorial]. *Am J Ind Med* 2013, 56:496–500. doi:10.1002/ajim.22083.

40. Reich JM: Neoplasia in the etiology of sarcoidosis [letter]. *Am J Med* 2013, 126(1):e17. doi:10.1016/j.amjmed.2012.05.031

41. Reich JM: Response to Dr. Michael Hodgson’s letter re. “Shortfalls in imputing sarcoidosis to occupational exposures.” *Am J Ind Med* 2013, 56(4):503–504. doi:10.1002/ajim.22149

42. Reich JM: Tissue confirmation of presumptive stage I sarcoidosis [editorial]. *J Bronchol Intervent Pulmonol* 2013 (in press)

## References

[B1] Erdal BS Clymer BD Yildiz VO Julian MW Crouser ED Unexpectedly high prevalence of sarcoidosis in a representative U.S. Metropolitan population Respir Med 2012 106 6 893 899 Epub 2012 Mar 13 10.1016/j.rmed.2012.02.007 22417737

[B2] Baldry PE Sutherland I Scadding JG Geographic variations in the incidence of sarcoidosis in Great Britain: a comparative study of four areas Tubercle 1969 50 211 232 5347533 10.1016/0041-3879(69)90048-8

[B3] Reich JM Johnson RE Incidence of clinically identified sarcoidosis in a Northwest United States population Sarcoidosis Vasc Diffuse Lung Dis 1996 13 173 177 8893388

[B4] Gribbin J Hubbard RB Le Jeune I Smith CJ West J Tata LJ Incidence and mortality of idiopathic pulmonary fibrosis and sarcoidosis in the UK Thorax 2006 61 11 980 985 10.1136/thx.2006.062836 16844727 PMC2121155

[B5] Hillerdal G Nöu E Osterman K Schmekel B Sarcoidosis:epidemiology and prognosis Am Rev Respir Dis 1984 130 29 32 6742607 10.1164/arrd.1984.130.1.29

[B6] Hennessy TW Ballard DJ DeRemee RA Chu CP Melton LJ 3rd The influence of diagnostic access bias on the epidemiology of sarcoidosis: a population-based study in Rochester, Minnesota, 1935–1984 J Clin Epidemiol 1988 41 6 565 570 10.1016/0895-4356(88)90060-1 3385457

[B7] Horwitz O Payne PG Wilbek E Epidemiology of sarcoidosis in Denmark Dan Med Bull 1967 14 178 182 6047311

[B8] Sartwell PE Edwards LE Epidemiology of sarcoidosis in the U.S. navy Am J Epidemiol 1974 99 4 250 257 4818715 10.1093/oxfordjournals.aje.a121609

[B9] Rybicki BA Major M Popovich J Jr Maliarik MJ Iannuzzi MC Racial differences in sarcoidosis incidence: a 5-year study in a health maintenance organization Am J Epidemiol 1997 145 234 241 10.1093/oxfordjournals.aje.a009096 9012596

[B10] Müller Quernheim J Gaede K Fireman E Zissel G Diagnosis of chronic beryllium disease within cohorts of sarcoidosis patients Eur Respir J 2006 27 6 1190 1195 10.1183/09031936.06.00112205 16540500

[B11] Hagerstrand L Linell F The prevalence of sarcoidosis in the autopsy material from a Swedish town Acta Med Scand 1964 176 425 171 173 10.1111/j.0954-6820.1964.tb05744.x 5884480

[B12] Reid JD Sarcoidosis in coroner’s autopsies: a critical evaluation of diagnosis and prevalence from Cuyahhoga County, Ohio Sarcoidosis Vasc Diffuse Lung Dis 1998 15 44 51 9572001

[B13] Reich JM Shortfalls in imputing sarcoidosis to occupational exposures Am J Ind Med 2013 56 496 500 10.1002/ajim.22083 22767391

